# Elderly Patients with Moderate Chronic Ischemic Mitral Regurgitation: Coronary Artery Bypass Grafting Alone or Concomitant Mitral Annuloplasty?

**DOI:** 10.1155/2019/1846904

**Published:** 2019-12-18

**Authors:** Qiang Ji, Yun Zhao, JinQiang Shen, YuLin Wang, Ye Yang, LiMin Xia, Kai Song, ChunSheng Wang

**Affiliations:** ^1^Department of Cardiovascular Surgery of Zhongshan Hospital Fudan University, Shanghai 180 Fenglin Road, Shanghai 200032, China; ^2^Shanghai Institute of Cardiovascular Diseases, 1609 Xietu Road, Shanghai 200032, China; ^3^Department of Cardiovascular Surgery of Xiamen Branch of Zhongshan Hospital Fudan University, 668 Jinhu Road, Huli District, Xiamen 510530, China

## Abstract

**Background:**

An increasing number of elderly patients with ischemic mitral regurgitation (IMR) are referred for coronary artery bypass grafting (CABG). However, data about the management of elderly patients with moderate IMR are scanty. This study evaluates the impacts of two surgical approaches (CABG alone or concomitant mitral annuloplasty (MAP)) on in-hospital and midterm outcomes, to attempt to determine an appropriate treatment option for elderly patients with moderate chronic IMR.

**Methods:**

All eligible patients over 65 years of age were included and were entered into either a MAP group (patients undergoing CABG plus MAP, *n* = 96) or a CABG group (patients receiving CABG alone, *n* = 104). Baseline and surgical characteristics were analyzed, and in-hospital and midterm outcomes between groups were compared after propensity score-matching (1 : 1).

**Results:**

Using propensity score-matching, 82 pairs of patients were successfully established in a 1 : 1 ratio. No significant differences between the two matched groups were found regarding surgical mortality (4.9% vs. 1.2%, *p*=0.173) and major postoperative morbidity. 150 patients (76 in the MAP group and 74 in the CABG group) received regular follow-up visit with the median duration of 37 months. Compared with the CABG group, the MAP group received a similar overall survival but a better recurrent MR-free survival (stratified log-rank *p*, 0.492 and < 0.001, respectively). Using Cox regression, the MAP group as compared with the CABG group did not affect midterm survival probability (propensity score-adjusted hazard ratio, 0.854; 95% confidence interval, 0.571–2.729, *p*=0.630). Additionally, patients in the MAP group had a significantly lower ratio of NYHA class III-IV at the latest follow-up by comparison with patients in the CABG group (19.7% vs. 35.5%, *p*=0.033).

**Conclusion:**

Compared with CABG alone, concomitant mitral annuloplasty is associated with improved midterm outcomes (including reduced IMR recurrence and improved cardiac functional class) but shares similar surgical mortality and major postoperative morbidity and may be a promising treatment option for elderly patients with moderate chronic IMR.

## 1. Introduction

With the acceleration of population aging, the advancement of surgical techniques, and the improvements in perioperative management, the current coronary artery bypass grafting (CABG) population has seen increased preponderances of aging and concomitant disease related to coronary artery disease [1–3]. Age is a risk factor for poor prognosis following surgical revascularization [4]. Compared with the young, elderly patients have a higher burden of surgical risk factors with reduced functional capacity and increased comorbidity conditions [5].

The development of ischemic mitral regurgitation (IMR) is associated with an increased risk of heart failure and mortality, and this risk increases with the severity of mitral regurgitation (MR) [6]. The optimal management of moderate chronic IMR (mitral valve procedure at the time of CABG or CABG alone) remains controversial. Although IMR recurrence occurs at follow-up, the mitral valve procedure at the time of CABG does reduce MR after surgery [7–12]. Although it may reduce MR at follow-up, CABG alone is not sufficient to eliminate MR [3].

Our previous study supported the concomitant mitral valve procedure instead of CABG alone for the treatment of moderate chronic IMR [13]. However, the subjects of our previous study included not just the elderly but the nonelderly. Given the increasing number of elderly patients with moderate chronic IMR referred for surgical revascularization, there is a need to determine which surgical approach (concomitant mitral valve procedure or CABG alone) is an appropriate treatment option for elderly patients with moderate chronic IMR. Unfortunately, the data about the management of elderly patients with moderate chronic IMR are scanty.

This study reviewed 200 elderly patients with moderate chronic IMR who underwent either CABG plus mitral annuloplasty or CABG alone in our institute and evaluated the impacts of the two surgical approaches on in-hospital and midterm outcomes, to attempt to determine an appropriate treatment option for elderly patients with moderate chronic IMR.

## 2. Methods

### 2.1. Study Protocol

This study protocol was approved by the Ethics Committee of *Zhongshan Hospital Fudan University* (no. B2018-118) and was consistent with the *Declaration of Helsinki*.

The etiology and severity of MR were determined by transthoracic echocardiography within 3 days before surgery. MR grade was defined as moderate (effective regurgitant orifice area <20 mm^2^, regurgitant volume < 30 ml, and regurgitant fraction < 50%) according to the literature [14]. MR was graded independently by two level 3 readers (discrepancies were resolved by a third reader as needed) as suggested by current guidelines with an integrated approach using all available parameters.

Consecutive patients over 65 years of age with moderate chronic IMR who underwent CABG plus mitral valve repair or CABG alone during the study period (from June 2010 to June 2017) were assessed for study eligibility by checking the following criteria: (1) prior myocardial infarction by electrocardiogram or regional wall motion abnormalities by echocardiography; (2) structurally normal mitral valve; and (3) sinus rhythm. Exclusion criteria were moderate or severe aortic stenosis or insufficiency, concomitant tricuspid annuloplasty, mitral valve organic pathology (prolapse, rheumatic, endocarditis, leaflet perforation, or extensive annular calcification), valve prosthesis, Marfan's syndrome, and unstable clinical conditions. Considering that patients undergoing complex mitral valve repair may not receive similar outcomes as patients undergoing mitral annuloplasty alone, patients who underwent concomitant leaflet and/or subvalvular procedure (including edge-to-edge, leaflet augmentation, secondary chordal cutting, and papillary muscle approximation) were excluded and patients who received mitral annuloplasty alone at the time of CABG were included. Additionally, considering that on-pump CABG alone accounted for less than 5% of CABG alone in our center and isolated off-pump CABG may not result in similar outcomes (especially in-hospital outcomes) as on-pump CABG alone, patients undergoing on-pump CABG alone or urgent conversion from off-pump to on-pump CABG during surgery were also excluded and patients receiving off-pump CABG alone were included.

All of the included patients were entered into a MAP group or a CABG group. Patients in the MAP group received mitral annuloplasty at the time of CABG aided by cardiopulmonary bypass, whereas those in the CABG group underwent off-pump CABG alone. Patients were regularly followed up at 3 and 6 months following surgery and thereafter at 6-month intervals. If patients developed symptoms or doubtful symptoms of MR or coronary artery disease at follow-up, clinic visits should be performed at the time.

Perioperative data were obtained from our institutional database and were reviewed using a standard data collection form. Follow-up data were obtained by clinic visit and/or telephone. Data collection was performed by trained staff (two people). The trained staff, however, were not informed of the purpose of this study. To reduce the effects of treatment selection bias and potential confounding, propensity score-matching (1 : 1) was used to adjust for differences in baseline characteristics. In-hospital and follow-up outcomes were compared between the two matched groups.

### 2.2. Outcomes

In-hospital outcomes consisted of surgical mortality and major postoperative morbidity. Major postoperative morbidity included myocardial infarction associated with CABG, low cardiac output, redo for bleeding, prolonged ventilation of more than 24 hours, stroke, renal failure requiring hemodialysis, and deep sternal wound infection. The definitions of surgical mortality and major postoperative morbidity referred to previous studies [15, 16]. Additionally, intra-aortic balloon pump (IABP) support, drainage during the first 24 h following surgery, blood transfusion, length of intensive care unit (ICU) stay, and length of postoperative hospital stay were also recorded.

Follow-up outcomes included all-cause death and IMR recurrence. All-cause mortality rather than cardiac mortality was chosen because it was the most robust and unbiased index which exempted us from misreading the cause of death with the subjective and sometimes inaccurate medical records. The occurrence of moderate or more MR determined by transthoracic echocardiography at follow-up was diagnosed as IMR recurrence. Additionally, cardiac function class (NYHA III-IV) was also recorded.

### 2.3. Surgical Procedures

In our institute, off-pump CABG surgery was introduced in 1998 and has been the first choice in patients who were referred for surgical revascularization alone over the years with an annual procedure volume of over 500 cases, whereas on-pump CABG was conducted in patients with severely impaired left ventricular function, deeply intramyocardial target vessels, previous cardiac surgery, or concomitant open-heart surgery. The decision to perform CABG alone or concomitant mitral annuloplasty was influenced by each patient's demographic and clinical profile (i.e., age, comorbidities such as chronic obstructive pulmonary disease and renal dysfunction, left ventricular endodiastolic diameter, and estimated surgical risks), but the choice was ultimately left to the discretion of the operating surgeon. All the procedures (either CABG alone or concomitant mitral annuloplasty) were performed by the same surgical team.

All surgical procedures were performed through a midline sternotomy. The internal mammary artery was harvested in a skeletonized or a pedicled fashion, and the left internal mammary artery grafting to the left anterior descending artery has been the first choice. Saphenous vein and radial artery were harvested with an open technique, and sequential or separate aortocoronary bypass grafting was performed in the remaining coronary arteries. The quality of anastomosis was assessed after grafting with a transit-time flow probe (Medistim Butterfly Flowmeter, Oslo, Norway) during the operation. The details of the off-pump and on-pump CABG procedures were consistent with those of previous studies [17, 18].

The mitral valve was exposed through a vertical transseptal approach along the right border of the foramen ovale, leaving the left atrial roof untouched. The size of the ring was determined after careful measurement of the height of the anterior leaflet and then downsizing. All included patients underwent mitral annuloplasty with a downsized rigid complete-ring (Edwards Physio rigid complete-ring). Rings were inserted using deep U-shaped simple horizontal sutures using Ethibond 2-0 (Ethicon, Inc, Somerville, NJ) or Ti-Cron 2-0 (Syneture, Norwalk, CT). The quality of mitral repair was determined by intraoperative transesophageal echocardiography immediately after discontinuation of cardiopulmonary bypass.

### 2.4. Statistics Analysis

Normally distributed continuous variables were expressed as the mean ± standard deviation, and nonnormally distributed continuous variables were expressed as median and interquartile range (IQR). Categorical variables were expressed as frequency distributions and single percentages. To control for measured potential confounders in the dataset, a propensity score (PS) was generated for each patient from a multivariable logistic regression model that was based on 19 characteristics (as listed in Table 1, including demographics variables, concomitant diseases, preoperative cardiac status variables, and number of grafts) as independent variables with surgical approach (CABG plus mitral annuloplasty vs. off-pump CABG alone) as a binary dependent variable. The discrimination power and calibration of the PS model were tested with the c-statistic and the Hosmer–Lemeshow goodness-of-fit. Two pairs of matched patients were obtained using the greedy-matching algorithm to implement nearest-neighbor 1 : 1, with a caliper width of 0.2 of standard deviation of the logit of the PS. The quality of the match was assessed by comparing selected pretreatment variables in propensity score-matched patients using the standardized mean difference.

Normally distributed continuous variables were compared between groups using Student's *t*-test before matching and paired *t* test after matching. Nonnormally distributed continuous variables were compared between groups with the *Wilcoxon* rank-sum test before and after matching. Categorical variables were compared between groups using *χ*^2^ test or Fisher's exact test (when appropriate) before matching and McNemar's test after matching. For the analysis of in-hospital outcomes in the matched population, McNemar's test with odds ratios (OR) and 95% confidence interval (CI) was used for categorical outcomes. The impacts of surgical approach (CABG plus mitral annuloplasty vs. off-pump CABG alone) as independent risk factors on surgical mortality and major postoperative morbidity were evaluated using conditional logistic regression analysis. Overall survival and recurrent IMR-free survival were estimated using the *Kaplan–Meier* method with the stratified log-rank test to compare the equality of the survival curves in the PS-matched population. The Cox regression model stratified on the matched pairs was used to estimate the PS-adjusted hazard ratio (HR) and 95% CI of midterm survival probability between the two matched groups. A two-sided *p* value less than 0.05 was considered statistically significant. Statistical analysis was performed with SPSS statistical package version 22.0 (SPSS Inc., Chicago, IL, USA).

## 3. Results

### 3.1. Study Population

**Figure 1:**
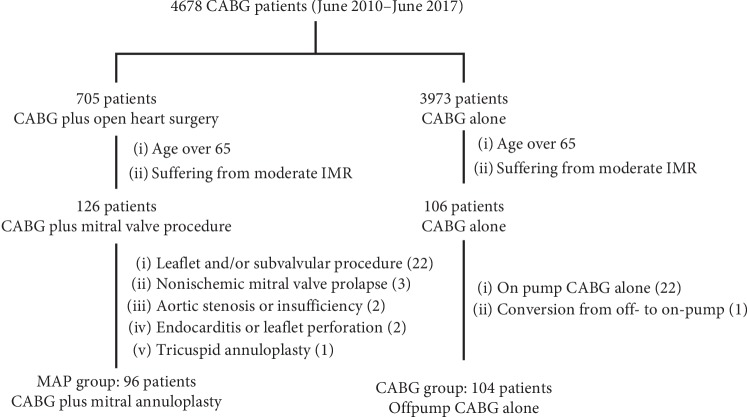
Flowchart of selected patients. CABG, coronary artery bypass grafting; MAP, mitral annuloplasty.

A total of 4678 consecutive patients underwent CABG alone (3973 patients, including 3845 off-pump CABG patients and 128 on-pump CABG patients) or concomitant open-heart surgery (705 patients) in our institute from June 2010 to June 2017. Of them, 232 patients over 65 of age who met the inclusion criteria were reviewed. As shown in Figure 1, 32 patients were excluded, which left 200 eligible patients for data analysis. Among 200 included patients, 96 patients who underwent mitral annuloplasty at the time of CABG were entered into the MAP group, and the remaining 104 patients who received isolated off-pump CABG were entered into the CABG group.

### 3.2. Baseline and Procedure Characteristics

Baseline characteristics are shown in Table 1. Patients in the MAP group as compared with the CABG group were younger (73.5 ± 2.2 years vs. 74.4 ± 2.6 years, *p*=0.009) and had lower left ventricular ejection fraction but higher left ventricular endodiastolic diameter (0.43 ± 0.14 vs. 0.47 ± 0.13, *p*=0.037, and 59.2 ± 6.6 mm vs. 56.8 ± 6.0 mm, *p*=0.008, respectively). In addition, compared with the CABG group, the MAP group had a greater effective regurgitant orifice area (*p*=0.016).

Characteristics of surgical revascularization are also shown in Table 1. All patients in the MAP group received on-pump technique, with mean cardiopulmonary bypass time of 95.9 ± 21.4 minutes and mean aortic cross-clamping time of 78.5 ± 13.7 minutes, whereas all patients in the CABG group underwent isolated CABG procedure without cardiopulmonary bypass. Patients in both groups received similar number of grafts (*p*=0.531).

In the MAP group, all patients received restrictive mitral annuloplasty with downsized rigid complete-rings (median size of 28 mm) in addition to CABG. Immediately after repair, transesophageal echocardiography showed no or trace MR in 88 patients (91.7%) and mild MR in 8 patients (8.3%), respectively. Transmitral pressure gradient of more than 5 mmHg was not found immediately after mitral annuloplasty.

### 3.3. Propensity Score-Matched Cohort

**Figure 2:**
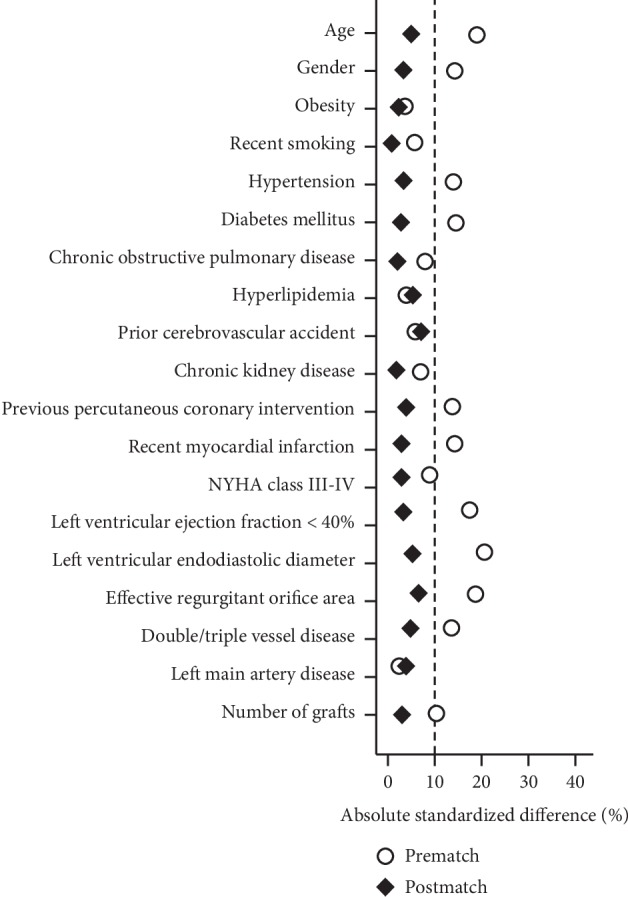
Absolute standardized differences for baseline covariates before and after matching. NYHA, New York Heart Association Functional Classification.

Bivariate analyses were conducted to examine differences in baseline characteristics between patients in the MAP group (*n* = 96) and those in the CABG group (*n* = 104). Propensity scores were then calculated using a multivariate logistic regression model based on the predefined 19 variables. The Hosmer–Lemeshow goodness for the model was 4.93 (*p*=0.726). Also, the model achieved good discrimination power with the receiver operating curve resulting in the area under the curve of 0.70 (95% CI, 0.61–0.80, *p*=0.027). Finally, 82 pairs of patients were successfully established in a 1 : 1 ratio. As shown in Figure 2, all the absolute standardized differences after matching were <10%, indicating an adequate balance.

As shown in Table 1, the two matched groups were comparable for baseline variables (including demographics variables, concomitant diseases, and preoperative cardiac status variables). Additionally, no significant differences were found between the two matched groups regarding surgical characteristics.

### 3.4. In-Hospital Outcomes in the Matched Population

No significant difference was found in surgical mortality between the two matched groups (4.9% vs. 1.2%, *p*=0.173). As shown in Table 2, there were no significant differences between the two matched groups regarding major postoperative morbidity, including MI associated with CABG, low cardiac output, prolonged ventilation, stroke, renal failure requiring hemodialysis, and deep sternal wound infection. No permanent neurological impairment was observed. Although the MAP group as compared with the CABG group had more drainage during the first postoperative 24 h and a higher ratio of blood transfusion (438 ± 93 ml vs. 261 ± 68 ml, *p* < 0.001, and 74.4% vs. 25.6%, *p* < 0.001, respectively), the two groups received a similar incidence of redo for bleeding (3.7% vs. 1.2%, *p*=0.620). Additionally, patients in the MAP group experienced a longer duration of ICU stay and longer length of postoperative hospital stay by comparison with patients in the CABG group.

The impacts of surgical approach (CABG plus mitral annuloplasty vs. off-pump CABG alone) on surgical mortality and major postoperative morbidity are shown in Table 2. Using conditional logistic regression analysis, surgical approach was not an independent risk factor for surgical mortality or major postoperative morbidity.

### 3.5. Follow-Up Outcomes in the Matched Population

**Figure 3:**
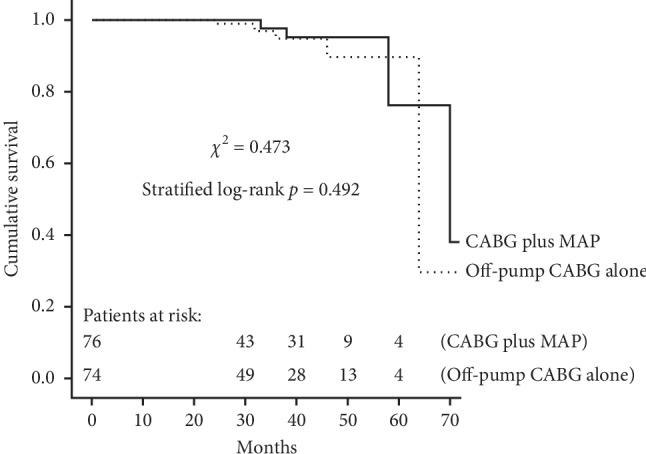
Kaplan–Meier analysis for overall survival in the matched population. CABG, coronary artery bypass grafting; MAP, mitral annuloplasty.

**Figure 4:**
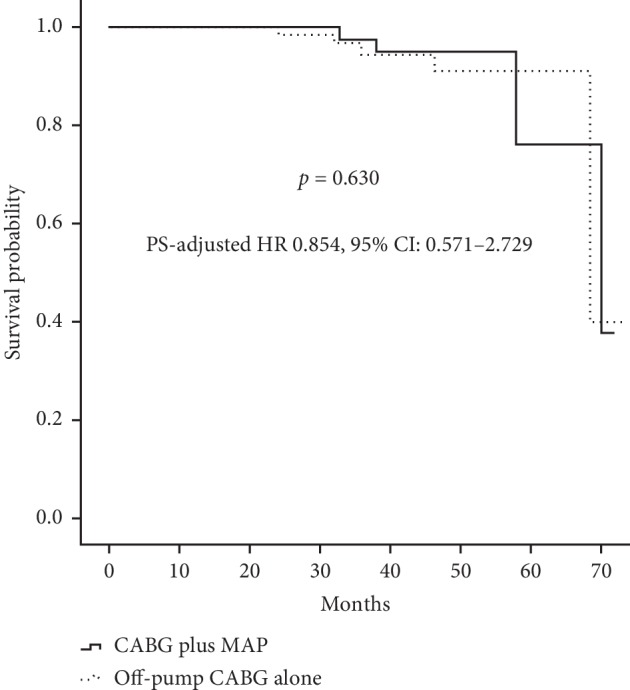
Survival probability using PS-adjusted Cox regression analysis. PS, propensity score; HR, hazard ratio; CI, confidence interval.

**Figure 5:**
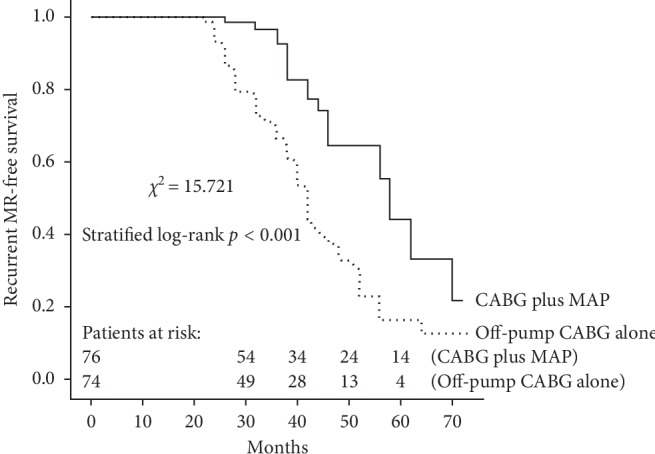
Kaplan–Meier analysis for recurrent IMR-free survival in the matched population.

A total of 150 patients (76 in the MAP group and 74 in the CABG group) received regular follow-up visit with a median duration of 37 months. Ten patients (4 in the MAP group and 6 in the CABG group) died during follow-up, with the cardiac mortality of 2.6% for the matched MAP group (*n* = 2) and 4.1% for the matched CABG group (*n* = 3). As shown in Figure 3, there was no significant difference between the two matched groups regarding overall survival (*χ*^2^ = 0.473, stratified log-rank *p*=0.492). Follow-up death among the matched population was modeled using Cox regression (Figure 4). The result showed that compared with off-pump CABG alone, CABG plus mitral annuloplasty was not associated with midterm survival probability (PS-adjusted HR, 0.854; 95% CI, 0.571–2.729, *p*=0.630). IMR recurrence occurred in 62 patients during follow-up (16 in the MAP group and 46 in the CABG group). As shown in Figure 5, Kaplan–Meier curves showed the MAP group as compared with the CABG group received a better recurrent MR-free survival (*χ*^2^ = 15.721, stratified log-rank *p* < 0.001). Additionally, patients in the MAP group had a significantly lower ratio of NYHA class III-IV at the latest follow-up by comparison with patients in the CABG group (19.7% vs. 35.5%, *p*=0.033).

## 4. Discussion

The results of midterm follow-up showed that although no benefit for midterm survival was observed, concomitant mitral annuloplasty as compared with CABG alone in the treatment of elderly patients with moderate chronic IMR received improved cardiac functional class and reduced IMR recurrence. In this study, CABG plus mitral annuloplasty as compared with off-pump CABG alone had a significantly lower ratio of NYHA class III-IV at the latest follow-up. This result suggested a significant benefit for midterm cardiac functional class after CABG plus mitral annuloplasty compared with CABG alone. Chan and colleagues [7] have conducted a prospective observational study (RIME trial) comparing CABG plus mitral valve procedure vs. CABG alone for 73 patients referred for CABG with moderate IMR and have reported an improved functional capacity with the CABG plus mitral valve procedure group. This evidence was consistent with our finding. Our finding was contradicted by the observations of Smith and colleagues [12], who reported similar improvement in functional capacity. This study showed that patients in the MAP group received a better recurrent MR-free survival by comparison with patients in the CABG group. A significant reduction of IMR recurrence may be an intuitive finding given patients in the MAP group undergoing mitral annuloplasty at the time of CABG. This result was in line with the evidence from previous studies [6–8]. Additionally, this study showed that the two surgical approaches received a similar overall survival, and CABG plus mitral annuloplasty as compared with off-pump CABG alone was not associated with midterm survival probability via Cox regression, suggesting that no benefit for midterm survival following concomitant mitral annuloplasty compared with CABG alone was observed. This result was in line with the evidence from previous studies [3, 6–8, 10–12].

Another important finding of this study is that compared with off-pump CABG alone, CABG plus mitral annuloplasty in the treatment of elderly patients with moderate chronic IMR received similar surgical mortality and major postoperative morbidity. Under the condition of comparable baseline characteristics and number of grafts, there were no significant differences between groups regarding surgical mortality and major postoperative morbidity. These results were further confirmed via multivariable logistic regression. So, our results suggested that compared with CABG alone, concomitant mitral annuloplasty did not bring about more in-hospital adverse events. Few studies have evaluated the impacts of CABG alone vs. concomitant mitral valve repair on in-hospital outcomes of elderly patients with moderate chronic IMR. A previous study [19], which compared the outcomes of 150 elderly patients with moderate IMR who underwent either off-pump CABG alone or CABG plus mitral valve procedure, has revealed that off-pump CABG group had statistically significant better early operative outcomes. This evidence was not in line with our study. The reason for this difference may have been the study population, regarding that elder patients who were included in this study had better left ventricular function and small left ventricular volume.

Despite similar incidence of in-hospital adverse events, the MAP group as compared with the CABG group received more drainage during the first postoperative 24 h, a higher ratio of blood transfusion, and longer length of ICU stay and postoperative hospital stay, suggesting patients undergoing CABG plus mitral annuloplasty received greater trauma and required longer duration of recovery by comparison with those undergoing off-pump CABG alone. This may be closely related to the use of cardiopulmonary bypass with cardiac arrest and prolonged operative time.

Results of this study showed that for treatment of elderly patients with moderate chronic IMR, concomitant mitral annuloplasty as compared with CABG alone reduced IMR recurrence and improved cardiac function and thus improved midterm outcomes. Also, concomitant mitral annuloplasty as compared with CABG alone received similar surgical mortality and major postoperative morbidity, suggesting concomitant mitral annuloplasty did not bring about more in-hospital adverse events. Due to the use of cardiopulmonary bypass with cardiac arrest and prolonged operative time, CABG plus mitral annuloplasty as compared with off-pump CABG alone may bring greater trauma and lengthen the duration of recovery, but did not significantly increase the incidence of in-hospital adverse events. Therefore, improved midterm outcomes coincided with similar surgical mortality and major postoperative morbidity, which may be in favor of CABG plus mitral annuloplasty as a promising treatment option for elderly patients with moderate chronic IMR.

There were several limitations to this study. First, it was only a single-center observational study involving a small sample size, which may influence the generalizability of its results. Also, this study showed that the incidence of adverse events was very low in both groups and also showed that no significant differences were found between the two approaches regarding surgical mortality and major postoperative morbidity, which may be related to a small sample size. A final determination would need a prospective, multicenter study involving a larger sample size. Second, all of the included patients in this retrospective study were not randomized into CABG plus mitral annuloplasty group or off-pump CABG alone group, which was associated with selection biases and influence on the interpretation of results. Although using propensity score-matching, confounds and selection biases among the two groups cannot be eliminated. Third, patients who underwent concomitant leaflet and/or subvalvular procedure were excluded from data analysis. Patients with complex mitral valve repair as compared with those with annuloplasty alone may receive more midterm benefits, which may be more likely to favor CABG plus mitral valve procedure as a promising treatment option for elderly patients with moderate chronic IMR. Finally, the duration of follow-up was relatively short. Although CABG plus mitral valve procedure was supported by our data for the treatment of moderate chronic IMR in elderly patients, further studies were warranted to substantiate and validate the evidence.

## 5. Conclusions

Compared with CABG alone, concomitant mitral annuloplasty is associated with improved midterm outcomes (including improved cardiac functional class and reduced IMR recurrence) but shares similar surgical mortality and major postoperative morbidity and may be a promising treatment option for elderly patients with moderate chronic IMR.

## Figures and Tables

**Table 1:** Baseline and procedure characteristics.

	Unmatched population			Matched population		
	MAP group	CABG group	*p*	MAP group	CABG group	*p*
	(*n* = 96)	(*n* = 104)		(*n* = 82)	(*n* = 82)	
Demographics						
Age (years)	73.5 ± 2.2	74.4 ± 2.6	0.009	73.8 ± 2.2	74.2 ± 2.5	0.278
Gender (female)	16 (16.7%)	23 (22.1%)	0.331	14 (17.1%)	17 (20.7%)	0.720
Obesity	12 (12.5%)	15 (14.4%)	0.691	10 (12.2%)	12 (14.6%)	0.832
Smoking history	38 (39.6%)	39 (37.5%)	0.762	33 (40.2%)	32 (39.0%)	0.873

Concomitant diseases						
Hypertension	64 (66.7%)	65 (62.5%)	0.538	54 (65.9%)	52 (63.4%)	0.923
Diabetes mellitus	35 (36.5%)	43 (41.3%)	0.479	31 (37.8%)	34 (41.5%)	0.804
Hyperlipidemia	23 (24.0%)	29 (27.9%)	0.527	20 (24.4%)	23 (28.0%)	0.761
COPD	11 (11.5%)	14 (13.5%)	0.757	10 (12.2%)	11 (13.4%)	0.815
Prior CVA	8 (8.3%)	12 (11.5%)	0.450	7 (8.5%)	9 (11.0%)	0.804
CKD	9 (9.4%)	12 (11.5%)	0.618	8 (9.8%)	9 (11.0%)	0.798

Preoperative cardiac status						
Previous PCI	22 (22.9%)	28 (26.9%)	0.513	19 (23.2%)	21 (25.6%)	0.875
Recent MI	43 (44.8%)	37 (35.6%)	0.184	38 (46.3%)	32 (39.0%)	0.550
NYHA III-IV	27 (28.1%)	21 (20.2%)	0.189	23 (28.0%)	18 (22.0%)	0.533
LVEF	0.43 ± 0.14	0.47 ± 0.13	0.037	0.44 ± 0.15	0.46 ± 0.14	0.379
LVEDD (mm)	59.2 ± 6.6	56.8 ± 6.0	0.008	58.7 ± 6.5	57.9 ± 6.1	0.418
ERO (mm^2^)	17 (13–18)	15 (11–17)	0.016	17 (13–18)	16 (12–17)	0.158

Extent of CAD						
2-vessel	14 (14.6%)	13 (12.5%)	0.667	10 (12.2%)	9 (11.0%)	0.807
3-vessel	82 (85.4%)	91 (87.5%)		72 (87.8%)	73 (89.0%)	
LM	30 (31.3%)	32 (30.8%)	0.941	26 (31.7%)	27 (32.9%)	0.867

Surgical data						
On-pump	96 (100%)	0		80 (100%)	0	
CPB time (min)	95.9 ± 21.4	0		96.0 ± 21.4	0	
ACC time (min)	78.5 ± 13.7	0		79.5 ± 13.8	0	
Number of grafts	3 (3, 3)	3 (3, 3)	0.531	3 (3, 3)	3 (3, 3)	0.724
Use of left IMA	95 (99.0%)	103 (99.0%)	0.955	81 (98.8%)	82 (100%)	0.316
Use of right IMA	0	5 (4.8%)	0.030	0	3 (3.7%)	0.080
Use of vein graft	94 (97.9%)	100 (96.2%)	0.465	81 (98.8%)	80 (97.6%)	0.560
Use of RA	3 (3.1%)	5 (4.8%)	0.723	3 (3.7%)	4 (4.9%)	1.000

MAP group, patients undergoing combined CABG and mitral annuloplasty; CABG group, patients undergoing off-pump CABG alone. BMI, body mass index; COPD, chronic obstructive pulmonary disease; CVA, cerebrovascular accident; CKD, chronic kidney disease; PCI, percutaneous coronary intervention; MI, myocardial infarction; AF, atrial fibrillation; NYHA, New York Heart Association; LVEF, left ventricular ejection fraction; LVD, left ventricular dysfunction; LVEDD, left ventricular endodiastolic diameter; LV, left ventricle; ERO, effective regurgitant orifice area; CAD, coronary artery disease; LM, left main trunk disease; CPB, cardiopulmonary bypass; ACC, aortic cross-clamping; IMA, internal mammary artery; RA, radial artery. Definition: obesity: body mass index of more than 30 kg/m^2^; recent MI: evidence of myocardial infarction within the last 30 days before surgery.

**Table 2:** In-hospital outcomes in the matched population.

Outcomes	MAP group (*n* = 82)	CABG group (*n* = 82)	Univariate analysis		Multivariate analysis	
			OR (95% CI)	*p*	OR (95% CI)	*p*
Surgical mortality	4 (4.9%)	1 (1.2%)	3.514 (0.754–8.291)	0.173	2.821 (0.883–6.214)	0.131
Major postoperative morbidity						
MI associated with CABG	1 (1.2%)	3 (3.7%)	0.425 (0.133–3.192)	0.311	0.618 (0.393–3.691)	0.364
Low cardiac output	7 (8.5%)	2 (2.4%)	3.733 (0.852–7.542)	0.167	2.873 (0.879–5.782)	0.106
Redo for bleeding	3 (3.7%)	1 (1.2%)	2.706 (0.713–6.203)	0.620	2.085 (0.816–5.737)	0.381
Prolonged ventilation	10 (12.2%)	5 (6.1%)	2.139 (0.697–5.659)	0.176	1.753 (0.789–5.732)	0.138
Stroke	4 (4.9%)	2 (2.4%)	2.051 (0.635–6.522)	0.682	1.573 (0.756–4.605)	0.405
RF requiring hemodialysis	3 (3.7%)	1 (1.2%)	3.076 (0.613–8.203)	0.620	2.332 (0.804–6.563)	0.239
DSWI	2 (2.4%)	1 (1.2%)	2.025 (0.780–5.817)	1.000	1.516 (0.803–5.528)	0.863
Other outcomes						
IABP support	6 (7.3%)	2 (2.4%)	3.158 (0.781–7.311)	0.277		
Blood transfusion	61 (74.4%)	21 (25.6%)	3.838 (2.185–8.513)	<0.001		
Drainage during 1^st^ 24 h (ml)	438 ± 93	261 ± 68		<0.001		
Length of ICU stay (h)	28.4 ± 7.5	17.5 ± 5.3		<0.001		
Postoperative hospitalized days	6.8 ± 1.8	5.5 ± 1.3		<0.001		

OR, odds ratio; CI, confidence interval; MI, myocardial infarction; CABG, coronary artery bypass grafting; RF, renal failure; DSWI, deep sternal wound infection; IABP, intra-aortic balloon pump; ICU, intensive care unit.

## Data Availability

The data used to support the findings of this study are available from the corresponding author upon request.
